# The Changes in Canine Parvovirus Variants over the Years

**DOI:** 10.3390/ijms231911540

**Published:** 2022-09-29

**Authors:** Xiangqi Hao, Yanchao Li, Xiangyu Xiao, Bo Chen, Pei Zhou, Shoujun Li

**Affiliations:** 1 College of Veterinary Medicine, South China Agricultural University, Guangzhou 510642, China; 2 Guangdong Provincial Key Laboratory of Prevention and Control for Severe Clinical Animal Diseases, Guangzhou 510642, China; 3 Guangdong Provincial Pet Engineering Technology Research Center, South China Agricultural University, Guangzhou 510642, China

**Keywords:** canine parvovirus, evolution, amino acid mutation, variants

## Abstract

Canine parvovirus (CPV-2) is one of the most important pathogens in dogs, and despite the continual development of vaccines against CPV-2, CPV-2 is still circulating in the canine population. The CPV-2a/2b/2c variant has replaced the original CPV-2 virus and seems to exhibit accelerated transmission. Although CPV-2 infection has been frequently reported, no studies have summarized information of CPV-2 variants currently circulating worldwide. To track the evolution of CPV-2, we downloaded and analyzed all VP2 sequences from the NCBI database (from 1978 to 2022). We found that CPV-2c shows a tendency to replace CPV-2a as the new dominant variant in Asia, South America, North America and Africa. Additionally, CPV-2c, which is prevalent in most regions of Asia, carries two special mutations in VP2, A5G and Q370R, and has become a dominant mutation with spillover already occurring. In conclusion, this summary of the types of global epidemic variants provides new insight into the evolution of CPV-2 and raises awareness for blocking the spread of this virus. The spread of Asian-derived CPV-2c urgently needs to be further under surveillance.

## 1. Introduction

Canine parvovirus disease is an acute, often fatal infectious disease characterized by vomiting and hemorrhagic enteritis caused by canine parvovirus (CPV-2) [1]. CPV-2, a member of the *Parvoviridae* family, is a nonenveloped DNA virus with an icosahedral structure. The virus has a diameter of approximately 25 nm and contains a linear single-stranded genome of approximately 5 kb [2]. The CPV-2 genome consists of two coding frames, with the 3′ end encoding nonstructural proteins, including NS1 and NS2, and the 5′ end encoding viral capsid proteins, including VP1 and VP2 [3]. Mature virus particles also contain VP3 generated by hydrolysis of VP2. The CPV-2 NS1 protein has remained relatively conserved during the process of virus evolution. It is a phosphorylated protein with multiple functions, such as inducing apoptosis and promoting oncolysis [4,5,6]. The VP2 gene is 1755 bp in length and encodes 584 amino acids. Additionally, the capsid is composed of 60 structural proteins, of which VP2 is the most important, accounting for 90% of the viral capsid and determining the host range, pathogenicity, antigenicity and tissue tropism of the virus [7,8].

CPV-2 appeared in the late 1970s and is considered a variant of feline panleukopenia virus (FPV) [9]. Initially, the VP2 protein of CPV-2 and FPV only differed by six amino acids [10]. However, with the spread of CPV-2, the rate of VP2 evolution reached 10^−4^ substitutions/site/year, spreading to multiple regions with the emergence of different variants in only two years after the first outbreak [11]. There are only a few amino acid changes from the original CPV-2 to CPV-2a, including M87L, I101T, A300G and D305Y. Phylogenetically, the CPV-2b subtype is caused by the N426D mutation at the base of CPV-2a and the CPV-2c subtype by the D426E mutation at the base of the CPV-2b subtype [10]. In summary, it is very important to monitor the evolution of VP2 and to be vigilant with regard to the emergence of new variants.

Due to the rapid evolution of CPV-2 and the continuous emergence of new variants, some immunized dogs still develop the disease [12,13,14,15]. Overall, understanding the molecular biological characteristics and epidemic status of CPV-2 is of great significance for the prevention and control of parvovirus disease. In 2017, we systematically analyzed the global epidemic of CPV-2 for the first time. In 2020, we found new subtype changes in China [16]. Here, we update the analysis of global CPV-2 evolution.

## 2. Results

### 2.1. Global Epidemic of CPV-2

**Figure 1:**
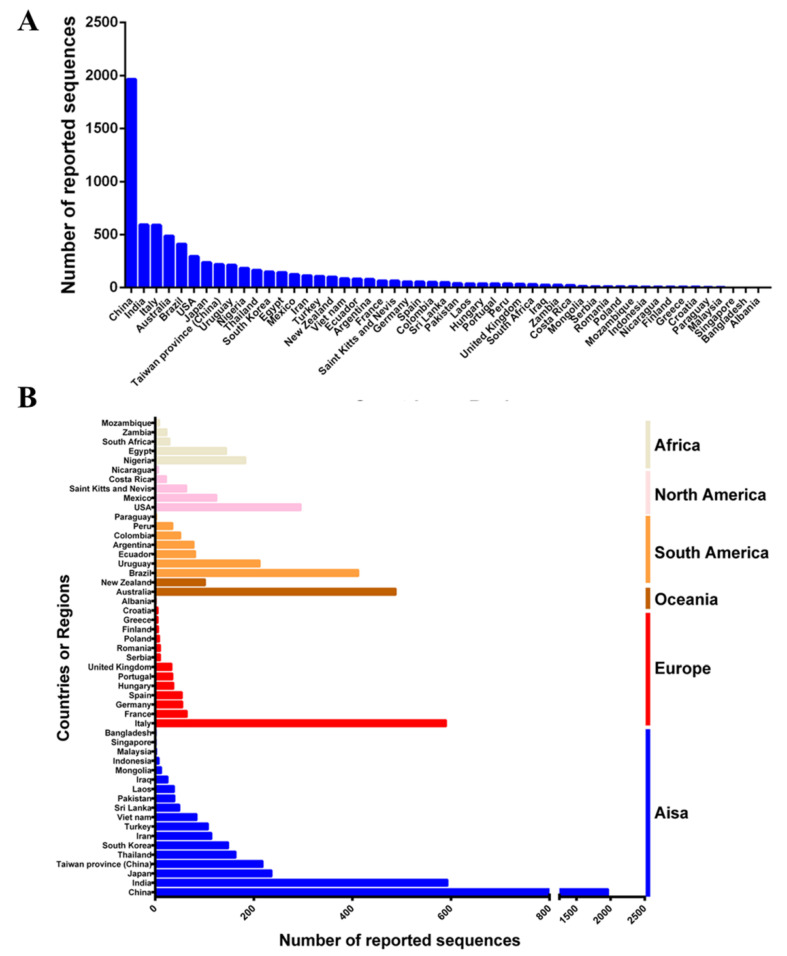
Distribution of the number of CPV-2 strains in different countries or regions: (**A**) sorted according to the number, (**B**) sorted according to the continent classification of the country or region.

Since CPV-2 was first reported in the 1880s, it has spread at an extremely fast rate around the world. As of June 2022, approximately 50 countries or regions have uploaded a CPV-2 sequence to GenBank. In total, 6211 VP2 sequences that contain information such as country or region and the time of sample collection were obtained. The most CPV-2 sequences are from China, followed by India, Italy, Australia, Brazil, the United States, and Japan (Figure 1A). Asia has provided the largest number of CPV-2 sequences (3431), followed by Europe (864), South America (753), Oceania (448), North America (413) and Africa (302) (Figure 1B). These results suggest that CPV-2 has been circulating globally.

### 2.2. Distribution of CPV-2 Variants

#### 2.2.1. Statistics of the Number of Variants of CPV-2a/2b/2c

**Figure 2:**
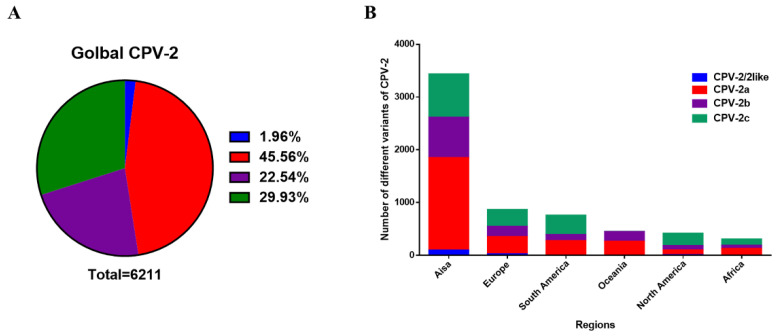
The number of CPV-2a/2b/2c variants. (**A**) Proportion of global CPV-2a/2b/2c. (**B**) Proportion of CPV-2a/2b/2c on each continent.

Among the 6211 VP2 sequences, CPV-2a has been reported to account for the vast majority (45.56%), and the proportion of CPV-2c (29.93%) surpasses that of CPV-2b (22.54%). Conversely, there are few CPV-2/2-like (1.96%) numbers, which are generally the source of vaccine strains or strains transmitted across species (Figure 2A). In addition to South America, North America has the largest number of CPV-2c variants, and CPV-2a accounts for most sequences in other continents (Figure 2B). These results suggest that CPV-2a has the most occurrences in the world.

#### 2.2.2. Overall Change of CPV-2a/2b/2c Globally

**Figure 3:**
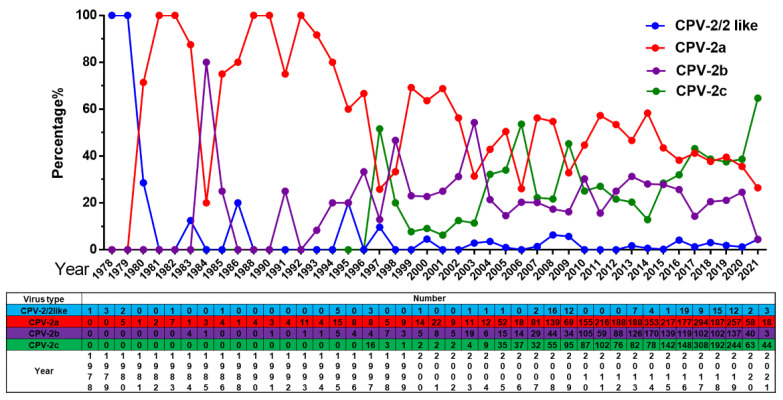
The number and proportion of CPV-2/2-like, CPV-2a, CPV-2b and CPV-2c in different years in the world.

Our analysis reveals a trend of common circulation of multiple CPV-2 variants worldwide, and these variants are undergoing dynamic changes. The original CPV-2 or CPV-2-like virus was replaced by CPV-2 variants after 1980, with a very low isolation rate. Prior to 2017, we reported that CPV-2a is the dominant variant in Asia and that CPV-2c is the dominant variant in Europe and Latin America [10]. In this update, the CPV-2c proportion has been increasing gradually, and it has replaced CPV-2a as the new dominant variant since 2020 (Figure 3). In addition, CPV-2b maintained a low epidemic status and peaked in 2003.

**Figure 4:**
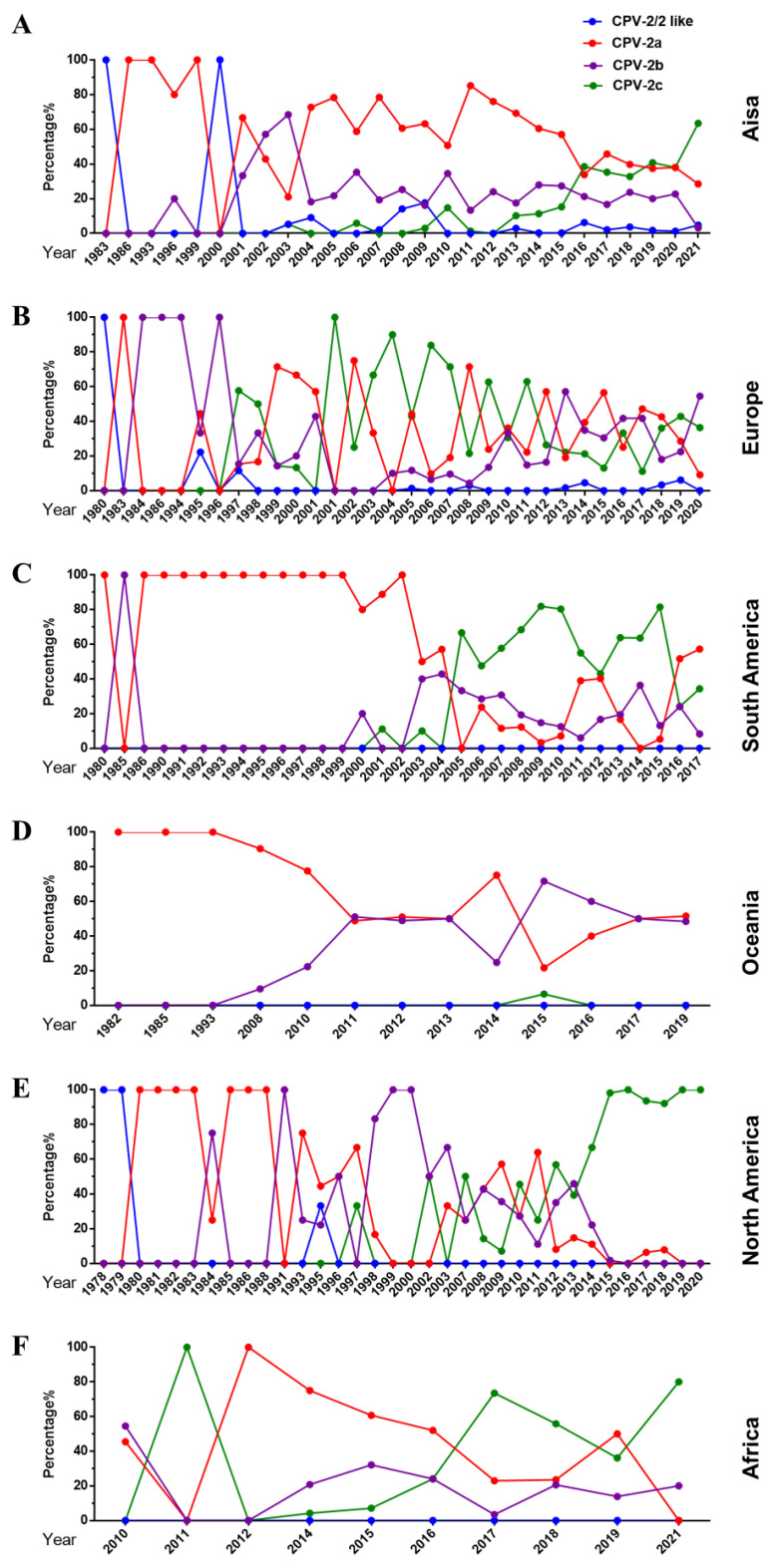
Changes in the proportion of CPV-2a/2b/2c on each continent. (**A**–**F**) The proportion of different variants on each continent over time.

The dynamic changes in the variant type of CPV-2 vary by region. In Asia, CPV-2a was prevalent as the dominant strain for a long time until it was replaced by rapidly growing CPV-2c in 2020 (Figure 4A). In Europe, CPV-2a/2b/2c are co-epidemic, accounting for a similar proportion, and the circulation rate of CPV-2/2-like is relatively low (Figure 4B). In 2004, CPV-2a replaced CPV-2c as the dominant variant in South America (Figure 4C). Regarding Oceania, there was a gradual change from CPV-2a to a co-endemic status for CPV-2a and CPV-2b (Figure 4D). Interestingly, before 2014, the predominant CPV-2a/2b/2c strains in North America changed repeatedly, and then CPV-2c became the main strain (Figure 4E). It is worth noting that CPV-2a replaced CPV-2c as the dominant variant after 2017 in Africa. In short, CPV-2c has become the dominant strain in Asia, South America, North America, and Africa. In Europe and Oceania, different variants are co-epdemic.

### 2.3. Analysis of Specific Amino Acid Mutation Sites in the VP2 Gene

**Figure 5:**
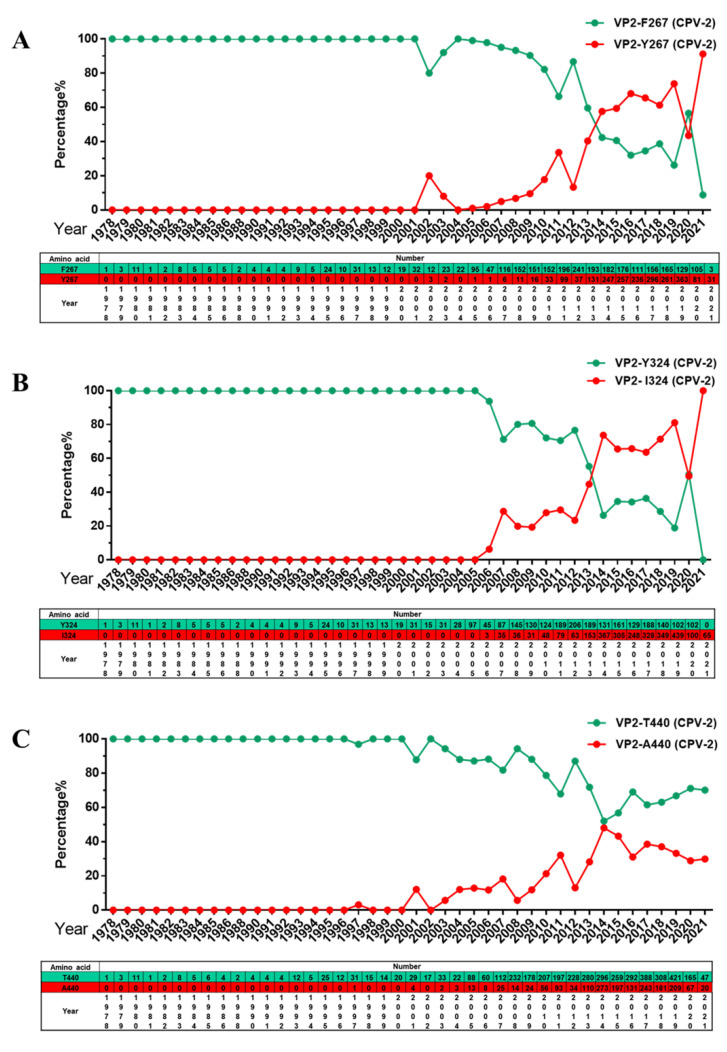
Analysis of global amino acid mutations at specific sites in the CPV-2 VP2 gene. (**A**) Percentage and number of amino acids at site 267 of VP2. (**B**) Percentage and number of amino acids at site 324 of VP2. (**C**) Percentage and number of amino acids at site 440 of VP2.

CPV-2 VP2 is the main component of the viral envelope. Several VP2 amino acids are related to antigenicity and host range [17], and antigenic drift may lead to cross-species transmission or vaccine failure [18,19]. We previously report that three mutant sites in VP2 (F267Y, Y324I and T440A) are of concern, which may be causes of vaccine failure [10]. In the present study, variation in these three amino acid residues was monitored, and the results showed that strains with 267Y and 324I mutations have continued to become dominant strains (Figure 5A,B). However, the frequency of 440A mutations did not surpass that of the original mutation 440T, which peaked in 2014 and then declined gradually thereafter (Figure 5C).

**Figure 6:**
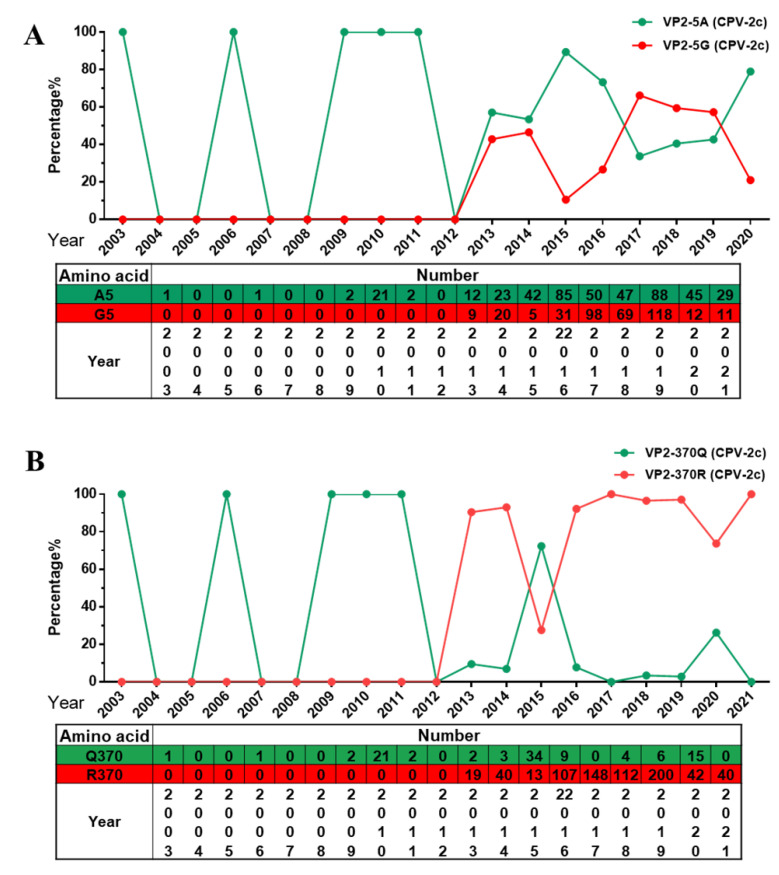
Analysis of amino acid mutations at specific sites in the Asian CPV-2c VP2 gene. (**A**) Percentage and number of amino acids at site 5 of VP2. (**B**) Percentage and number of amino acids at site 370 of VP2.

At present, CPV-2c has become the dominant variant in most regions of the world [20,21]. In this study, we mainly analyzed amino acid mutations in the VP2 gene of CPV-2c in Asia (includes 821 items). Interestingly, the two previously reported mutation sites, A5G and Q370R [16], have become unique mutations carried by CPV-2c. These two mutations appear to have been positively selected during replication in animals. A5G and Q370R first appeared in CPV-2c in 2013. Through analysis of the A5G mutation, we found that it has the characteristics of becoming the dominant CPV-2c (Figure 6A). Furthermore, the amino acid at 370 of VP2 has changed from Q to R, rendering this strain absolutely dominant (Figure 6B). We found that the two mutation sites A5G and Q370R occur in CPV-2c at the same time and the number of this strain has increased gradually, suggesting that this double-mutant strain may spread further. However, there are only a few sequences of this variant outside of Asia, only found in Europe (Italy and Romania), Africa (Nigeria and Egypt).

### 2.4. The Q370R Locus on the Surface of the VP2 Protein Pocket

**Figure 7:**
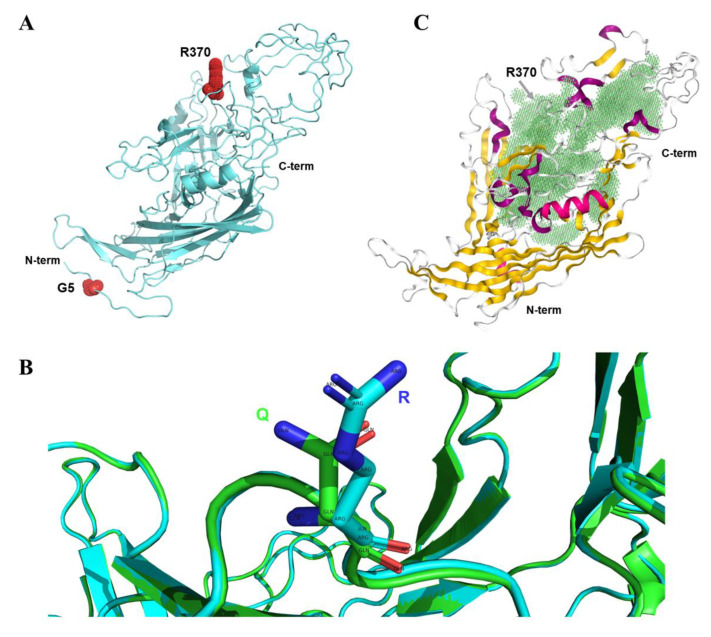
Three-dimensional structure analysis of the mutant VP2 protein. (**A**) VP2 structure of the A5G and Q370R double mutations. The mutated amino acid sites are labeled using red spheres. (**B**) Comparison of the three-dimensional protein structures of the prototype and the Q370R mutant VP2 using PyMOL software. (**C**) The protein-binding pocket of VP2 is predicted. The cavity of the pocket is filled with green color.

In this study, we constructed the 3D protein structure of A5G and Q370R mutant VP2, revealing that residue 5 is located at the N-terminus and residue 370 on one of the loops of VP2 (Figure 7A). Previous studies have suggested that the Q370R mutation may affect the host range and interaction between the host DNA and VP2 protein [22]. We compared the 3D protein structure of the prototype and the Q370R mutant VP2 and found that the local conformational changes were not significant (Figure 7B). Next, we predicted the position of the protein-binding pocket of VP2. Interestingly, residue 370 is located at the edge of the pocket (Figure 7C), and mutation at this site may have an important impact on specific protein interactions. In summary, the presence of the Q370R mutation may affect the binding ability of VP2. Nonetheless, the consequences of the A5G mutation are still unclear and worthy of further study.

## 3. Discussion

CPV-2 has been continuously transmitted for more than 40 years since its appearance. This virus can cause hemorrhagic diarrhea in dogs and myocarditis in puppies. At present, the disease is trending in the direction of acute onset and high mortality, and it is a major infectious disease that harms canine health [23]. The initial purpose of this study was to investigate the molecular characteristics of the currently prevalent CPV-2 variants. After analysis, we found that 50 countries or regions have deposited CPV-2 sequences in GenBank, at least twice the number of countries and regions reported in 2017 [10]. We found 6221 strains of CPV-2, including the location and date of collection, approximately three times the number in our previous study in 2017 [10]. This indicates that the virus is gaining more attention and suggests that it is in a state of continuous epidemic spread. Among these countries, China has the largest number of CPV-2 sequences, constituting a good model for studying the genetic evolution and transmission of this virus. There are more sequences uploaded from China, and one of the reasons may be that China is a large country and the number of dogs depends on the number of people.

In this analysis, CPV-2a was shown to be a major epidemic variant in the past and has certain advantages in most regions of the world. In addition, the circulation ratio of CPV-2/2-like is low, which may stem from the use of vaccination. However, based on the temporal dynamic analysis of the number of the variant, it is interesting to note that CPV-2c has been the mainstream variant for nearly 20 years from 1997 to 2017. After 2017, its number increased rapidly and replaced CPV-2a as a new mainstream variant (Figure 3). Previous studies suggested that Asian-derived CPV-2c has spread to Europe and Africa [20]. We speculate that CPV-2c turnover events may also occur in Europe and that a new variant may spread further in a very short period of time in the absence of intervention. The increase in CPV-2c in China may be a good example [16].

The global rapid growth of CPV-2c has attracted our attention because CPV-2c has previously grown rapidly in China, and this Asian-derived CPV-2c has already exhibited spillover [16,24]. We speculate that, due to its continuous growth, CPV-2c has gained some advantages during evolution. Therefore, we conducted a summary analysis of the VP2 sequence of CPV-2c in the Asian region, and the results showed that the newly emerged A5G and Q370R mutation sites have been preserved in the process of evolution. A5G was first reported in China in 2015, though its potential functional consequences remain to be determined [25]. Q370R first appeared in CPV-2a isolated from giant pandas in Sichuan, China, and subsequently became the dominant mutation site of CPV-2c [22]. Moreover, the proportion of Q370R mutations has increased yearly (Figure 6B). The A5G mutation is located at the N-terminus of VP2, and Q370R is located around the protein-binding pocket. Although local conformational changes are not obvious, these mutations may lead to changes in host range and protein-binding capacity. In addition, the F267Y and Y324I mutations identified in a previous report became the absolute dominant mutation at a very early stage, as confirmed in this surveillance [10,16]. It is necessary to use reverse genetics technology to study the effect of VP2 mutation sites on the virus and the host. In addition, monitoring of the CPV-2 variant is necessary. Several immunologically based assays have been established, including the blood agglutination test or blood agglutination inhibition test [26,27], the colloidal gold test method [28], immunofluorescence and immunoperoxidase [29], enzyme-linked immunosorbent assay [30,31], and so on. Although there are significant antigenic differences between the variants of CPV-2, they still have considerable homology, so the above methods showed deficiencies in distinguishing between variant types. Fortunately, several assays have been established to distinguish CPV-2 variants, and they are mainly based on PCR methods, including minor groove binder probe assays [32], fluorescence melting curve analysis [33], SimpleProbe real-time PCR assay [34], multiplex TaqMan real-time PCR method [35], high-resolution melting curve analysis [36], amplification refractory mutation system PCR [37], etc. This type of assay often requires a short detection time and is highly sensitive. The methods described above allow for the reliable identification of CPV-2 variants and have been applied for diagnostic and epidemiological studies on the distribution of variants in different countries or regions.

Vaccine immunization is key to disease prevention. However, CPV-2c breakthrough infection often occurs [12,13,14,15]. There may be several reasons for vaccine failure. Among them, interference of maternal antibodies may be an important factor [38], or coinfection with other viruses may lead to immunosuppression in dogs [39,40,41]; the greatest probability is a change in the antigenicity of the virus. At present, the vaccine commonly used in China is still original CPV-2 or CPV-2b [16], and vaccination failure is related to a large extent to the poor protection of CPV-2c [15,42]. The development of a CPV-2 vaccine requires an investigation of the main variants currently prevalent in the canine population. A vaccine with a mixture of different variant strains may also be a viable option. Whether this vaccine can provide complete protection against 5G and 370R mutant strains is worth studying. In summary, 5G and 370R mutant strains may become a new CPV-2c subvariant. Further functional studies of these mutants are needed.

## 4. Materials and Methods

### 4.1. Sequence Data Collection

To analyze the current global distribution of CPV-2 variants, the CPV-2 sequence (6686) as of August, 2022, was retrieved and downloaded from the NCBI database (“canine parvovirus”—Nucleotide—NCBI (nih.gov)) (accessed on 1 August 2020). After screening, we obtained 6211 VP2 sequences originating from dogs, which contained information such as country or region and the date of sample collection. The sequences were further classified according to the country or region where the samples were collected and were sorted according to the date of collection (Supplementary Materials).

### 4.2. Sequences and Amino Acid Analysis

The full-length and partial coding sequence (CDS) nucleotide sequences of VP2 were aligned using MEGA7 (Version 7.0.26) and BioEdit (Version 7.0.9.0) and then translated into amino acid sequences for further alignment. Finally, the amino acid mutations at the key sites were predicted and then summarized using Microsoft Excel.

### 4.3. Graph

GraphPad Prism (Version 6.01) was used to plot the amino acid mutation chart, pie chart, column chart, and line chart in this study.

### 4.4. D Modeling Prediction of the VP2 Structure

The 3D structure of VP2 was predicted and constructed on SWISS-MODEL based on the homology modeling method (https://swissmodel.expasy.org/). Visual editing of the structure was carried out using PyMOL software (Version 2.5.0). This structure was based on the VP2 sequence of a CPV-2c strain from Guangzhou, China (GenBank: KY937662) in our previous etiological investigation [16]. In addition, the VP2 protein pocket was predicted using the Yinfotek platform (https://cloud.yinfotek.com/) based on algorithms such as GHECOM.

## 5. Conclusions

This analysis shows that CPV-2c has replaced CPV-2a to become the dominant strain. The A5G and Q370R mutations have been preserved during evolution of CPV-2c. Moreover, VP2 sequences from China were screened for specific amino acid mutations that were used as markers of evolution.

## Data Availability

The canine parvovirus sequence information used in this study is from the NCBI public database and all data generated or analyzed during this study are included in this manuscript.

## Supplementary Materials

The following supporting information can be downloaded at: https://www.ncbi.nlm.nih.gov/nuccore/?term=%22canine+parvovirus%22.
